# Association between migration and cognitive status among middle-aged and older adults: a systematic review

**DOI:** 10.1186/s12877-017-0585-2

**Published:** 2017-08-17

**Authors:** Hanzhang Xu, Yinan Zhang, Bei Wu

**Affiliations:** 1 Duke University School of Nursing, Durham, NC USA; 2 Duke Global Health Institute, Duke University Medical Center, Durham, NC USA; 3 Chinese Center for Health Education, Beijing, China; 4 New York University Rory Meyers College of Nursing, New York, NY USA

**Keywords:** Cognition, Memory disorder, Dementia, Emigration, Immigration

## Abstract

**Background:**

This study aimed to synthesize the current literature examining the association between migration and cognitive function among middle-aged and older adults.

**Methods:**

We used the PRISMA as a guideline for this systematic review and searched the following databases: PubMed, CINAHL, EMBASE, and Global Health.

**Results:**

Twenty-five published studies were included. Twenty-two studies were focused on international migrants, while only 3 studied internal migrants. Fourteen studies were conducted in the United States, followed by UK (*n* = 2), Israel (*n* = 2), India (*n* = 2) and other countries like Canada and Australia. Some studies showed that middle-aged and older migrants demonstrated poorer cognitive function comparing to non-migrants in hosting places; while other studies indicated no association between migration and cognitive function. A higher level of acculturation was associated with better performance on cognitive function tests among migrants.

**Conclusion:**

It is unclear how or whether migration and cognitive function are related. The quality of current literature suffered from methodological deficiencies. Additional research is needed to examine the linkages using more comprehensive measures of migration and cognitive function.

## Background

Migration, defined as the geographic movement of people across a specified boundary for the purpose of establishing a new permanent or semi-permanent residence, is one of the three demographic components (i.e. birth, death, and migration) used to assess population changes [1]. Increasing migration both within countries and internationally has been observed globally [2]. It is estimated that the number of international migrants reached 232 million in 2013, and another 740 million were internal migrants [2]. Overall, migrants move from less developed areas (e.g. rural setting, developing countries) to more developed areas (e.g. urban setting, developed countries) [2].

An increasing number of migrants are entering into old age, and many of them suffer from deterioration of health outcomes in later life, including cognitive decline [3, 4]. Understanding the association between migration and cognitive function would provide better knowledge of risk factors related to cognition and help develop strategies and programs to promote healthy aging among the migrant populations.

**Fig. 1:**
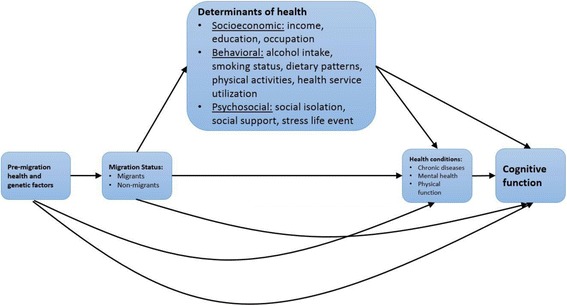
Potential linkages between migration and cognitive function

Migration is a major life event and its associated changes, including changes in socioeconomic status (SES), lifestyle, and environment, may have a significant impact on health status in later life (see Fig. 1) [5, 6]. A number of studies have shown that adulthood SES such as education, income, and occupation are protective factors in cognitive decline [7–10]. Migration may result in an improvement in an individual’s socioeconomic status (SES). For example, rural-to-urban migrants tend to have more exposure to education opportunities, which may positively influence their cognitive function in late life [11]. Moreover, these positive effects may lead to improvement in access to care and better management of chronic conditions, which could contribute to better cognitive function over time [12].

Behavioral changes have been observed in the migrant population as well. When migrating from a rural to an urban setting, and from a low and middle- income country to a high-income country, westernized life styles are often adopted [13–15]. Migrants are thought to be adopters of high-risk lifestyle that include calorie-intensive dietary patterns and physical inactivity [16]. They are often exposed to mechanized or sedentary employment and tobacco use [17–19]. These high-risk lifestyles are associated with the development of chronic diseases, including cardiovascular diseases, and poor cognitive function [20–22].

In addition, migrants often experience stressors during and after the migration process [23]. The separation from family is likely to be associated with reduced level of social support and size of social network. The perceived discrimination and a lack of sense of belonging in their destination may contribute to social isolation and development of depressive symptoms [24, 25], which can affect late-life cognitive function [26].

Currently, it remains to be unknown whether adult migrant population has better cognitive function compared to individuals who do not migrate. This significant gap in our knowledge is concerning given the rapidly increasing aging and migrant population. Therefore, we conducted a systematic review in order to examine the association between migration and cognitive function and provide direction for future research. If there is an association, this finding will have potential implications for clinical practices as well as health policies.

## Methods

**Fig. 2:**
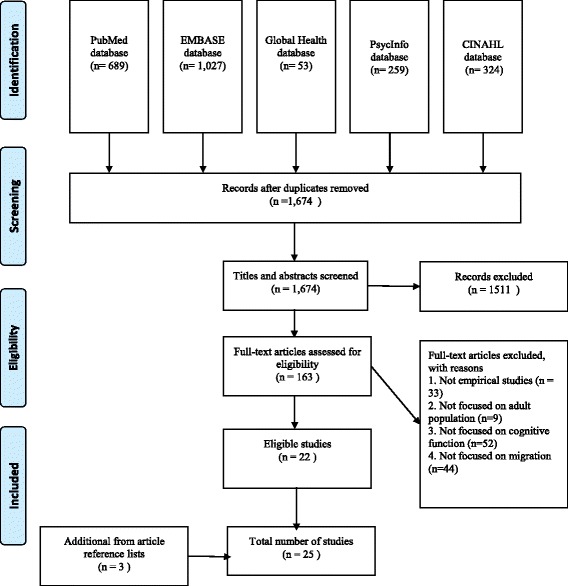
Selection of eligible articles

This literature review was conducted using the Preferred Reporting Items for Systematic Reviews and Meta-Analyses as a guideline [27]. We searched PubMed on January 19, 2016 using the following terms: (“Memory Disorders” [Mesh] OR “Cognition” [Mesh] OR “Cognition Disorders” [Mesh] OR “Dementia” [Mesh]) AND (“emigration and immigration”[MeSH Terms] OR (“emigration” [All Fields] OR “immigration” [All Fields]) OR “emigration and immigration” [All Fields]) OR (“emigrants and immigrants” [MeSH Terms] OR (“emigrants” [All Fields] OR “immigrants” [All Fields]) OR “emigrants and immigrants” [All Fields]) OR “residential mobility” [MeSH Terms] OR “transients and migrants” [MeSH Terms] OR “migration” [All Fields] OR “migrant*” [All Fields] OR “mass-migration” [All Fields] OR “Human Migration” [Mesh] OR “Population Dynamics” [Mesh]). Four additional datasets (EMBASE, Global Health, PsycInfo, and CINAHL) were also searched using similar terms. The search procedures are described in the Fig. 2. Results were limited to human subjects and English language, which yielded a total of 1674 articles. We applied two rounds of exclusionary criteria to identify non-qualifying articles that did not involve the adult population, did not examine cognitive function, did not focus on migration, or were not empirical studies. We first excluded 1511 articles by reviewing titles and abstracts, and 163 articles remained. We further eliminated 141 articles by scanning full text and we retained 22 articles. To these were added 3 articles culled from article reference lists, resulting in a total of 25 studies.

Two independent reviewers assessed the eligibility of each articles, extracted the information, and evaluated the quality of each study based on a set of established criteria. Disagreements between the two independent reviewers were discussed and resolved with the third reviewer. Information extracted from each eligible article included author and date of publication, study design, sample and location, outcome measures, and key findings. Given the heterogeneity in study design, definitions of migration, and outcome measures used, completing a meta-analysis was considered inappropriate.

## Results

### Characteristics of the eligible studies

A total of 25 articles were included in this review (Table 1), of which 22 were focused on international migrants, while only 3 studied migrants within their home country. Fourteen studies were conducted in the United States, followed by UK (*n* = 2), Israel (*n* = 2), and India (*n* = 2). Other study sites included Australia (*n* = 1), Denmark (*n* = 1), Canada (*n* = 1), the Netherland (*n* = 1), and Belgium (*n* = 1). Among the 14 studies conducted in the United States, 8 of them were focused on Mexican Americans. The majority of the studies were community-based, but two studies included participants from both community and institutions [28, 29].

**Table 1:** Characteristics of eligible studies of migration and cognitive status

Author, publication year (reference number)	Sample	Study design	Cognitive Measures	Migration Measures	Primary Covariates	Key findings
Adelman, S., et al. (2011) (28)	436 aged ≥60 living in UK (218 African-Caribbean immigrants, 218 Whites UK-born) Community and institutional setting	Cross-sectional study	Cognitive status: 1. CAMDEX 2. MMSE Dementia: ICD-10 DSM-IV NINCDS-ADRDA NINDS-AIREN	Country of birth	Age, Education, Gender, Marital status	Compared to native Whites, the prevalence of dementia was significantly higher in the African-Caribbean immigrant group
Kave, G., et al. (2008) (29)	814 older Jewish population in Israel aged ≥75 (138 Asian/African immigrants, 276 European/American immigrants, 400 nonimmigrants) Community and institutional setting	Longitudinal study follow-up period: up to 12 years	Cognitive status: 1. The Katzman et al. test 2. MMSE	Country of birth, Age at immigration	Age, Education, Occupation, Income, Number of language speak	No significant differences were found in cognitive function among Asian/African immigrants, European/American immigrants, and nonimmigrants Age at immigration was not significantly associated with cognitive function. Multilingualism was significantly associated with better cognitive status
Chertkow, H., et al. (2010) (30)	632 patients with probable AD from a Memory Clinic in Montreal, Canada (158 immigrants and 474 nonimmigrants) Clinical setting	Longitudinal study follow-up period: up to 1 year	Cognitive status: 1. MMSE Dementia: AD: NINCDS-ADRDA MCI diagnosis: clinical evaluation	First language Born in Canada	Age, Education, Gender, Occupation	No significant differences were found regarding age of dementia diagnosis between nonimmigrants and immigrants. No significant differences were found regarding age of symptom onset between nonimmigrants and immigrants Compared to immigrant unilinguals, native English unilinguals were likely to be diagnosed at a later age. No significant differences were found regarding rate of cognitive decline between nonimmigrants and immigrants
Zahodne, L. B., et al. (2014) (31)	1067 Spanish speaking immigrants in the U.S. aged ≥65 from Dominican Republic, Puerto Rico, or other Caribbean countries Community setting	Longitudinal study, follow-up period: up to 23 years	Cognitive status: 1. SRT 2. 15 item version of the Boston Naming Test 3. WAIS-R 4. Identities and Oddities subtest of the Mattis Dementia Rating Scales 5. the total number of words beginning with P, S, or V 6. Color Trails Test Dementia: DSM-III	Country of birth Age at immigration	Age, Education, Gender, Cohort, Bilingualism, Fluency in English	Immigrants from Dominican Republic/Puerto Rico showed poorer initial performance on memory and language tests Longer stay in the US was associated with better language function at baseline Country of origin and time in the US were not associated with rate of change in any cognitive domain
Hill, T. D., et al. (2012) (32)	2734 aged ≥65 Mexican Americans in the U.S, (first and second generation) Community setting	Longitudinal study, follow-up period: up to 14 years	Cognitive status: 1. MMSE	Country of birth Age at immigration	Age, Education, Gender, Financial strain, English proficiency, Depression, Smoking and drinking behaviors, Physical functioning, Chronic diseases (diabetes, stroke, hypertension, heart attack)	Compared to U.S born Mexican American, immigrants who migrated to the U.S in their middle age showed better cognitive function at baseline. Compared to U.S born Mexican American, male immigrants who migrated to the U.S in their middle age showed slower rate of cognitive decline.
Black, S. A., et al. (1999) (33)	2853 aged ≥65 Mexican Americans (first and second generation) in the U.S. Community setting	Cross-sectional study	Cognitive status: 1. MMSE	U.S.-born/foreign born	Age, Education, Gender, Literacy, Depression, Marital status, Physical functioning, Chronic diseases (diabetes, stroke, hypertension)	Immigrants were more likely to experience severe cognitive impairment (MMSE < 18) compared to U.S-born Mexican Americans Individuals that were interviewed in Spanish were less likely to be cognitively impaired or severe cognitively impaired.
Nguyen, H. T., et al. (2002) (34)	1759 aged ≥65 Mexican Americans in the U.S. (first and second generation) Community setting	Longitudinal study follow-up period: 5 years	Cognitive status: 1. MMSE	U.S. born/foreign born	Age, Education, Gender, Marital status, Household composition, Chronic diseases (diabetes, stroke, hypertension), Depression, Hearing and vision difficulties	No significant differences in cognitive decline (Decline to MMSE ≤17, Decline at least three points) were found between U.S. born Mexican Americans and Mexican immigrants to the U.S.
Sheffield, K. M. and M. K. Peek (2009) (35)	3050 aged ≥65 Mexican Americans in the U.S (first and second generation) Community setting	Longitudinal study follow-up period: up to 9 years	Cognitive status: 1. MMSE	U.S. born/foreign born	Age, Education, Gender, Marital status, Chronic diseases (diabetes, stroke, hypertension), Depression, Neighborhood characteristics, Socioeconomic disadvantages	No significant differences in both baseline cognitive function and cognitive decline were found between U.S. born Mexican Americans and Mexican immigrants to the U.S. Respondents living in economically disadvantaged neighborhood were more likely to have faster cognitive decline than those living in advantaged neighborhood
Lawton, D. M., et al. (2015) (36)	81 (55 AD, 26 VaD) Mexican Americans in the U.S. (first and second generation) aged ≥60 Community setting	Cross-sectional study	Cognitive status: 1. 3MSE 2. SEVLT 3. The Spanish English Neuropsychological Assessment Scale 4. The Informant Questionnaire on Cognitive Decline in the Elderly Dementia: AD: NINCDS-ADRDA VaD: NINDS-AIREN Age of dementia diagnosis	U.S. born/foreign born	Bilingualism	No significant differences were found regarding the proportion of types of dementia among bilingual U.S born Mexicans, bilingual Mexican immigrants, monolingual U.S born Mexican, and monolingual Mexican immigrants. No significant associations were found between immigrant status and age of dementia onset No significant interactions were found between bi/monolinguals and immigrant status on age of dementia diagnosis
Mejia, S., et al. (2006) (37)	12,008 aged ≥65 (3398 Spanish-speaking U.S. immigrants, 8610 Mexicans) Community setting	Cross-sectional study using several large datasets	Cognitive status: 1. Reduced Mini-Mental State Examination 2. Cross-cultural cognitive examination 3. Minineuropsi 4. MMSE 5. Modified Telephone Interview Cognitive Scale	Mexicans: 1. The Cognitive Factors Survey in Mexico City’s Elderly Population, 2. Salud, Bienestary Envejcimiento, 3. The Mexican Health and Aging Study Immigrants: 1. Hispanic Established Population for the Epidemiological Study of the Elderly 2. Health and Retirement Survey and Assets and Health Dynamics Among the Oldest Old Survey	Age, Education, Gender, Marital status, Physical functioning,	The prevalence estimates of cognitive impairment were similar among immigrants and Mexicans The prevalence of cognitive and functional impairment for immigrants were as least twice as high as for Mexicans
Raina, S. K., et al. (2014) (38)	2000 aged ≥60 older adults in India (500 urban residents, 500 rural residents, 500 migrants, 500 tribal population) Community setting	Cross-sectional study	Cognitive status: 1. Hindi Mental State Examination (HMSE) Dementia: Clinical evaluation	N/A	N/A	Urban residents had a higher prevalence of cognitive impairment than rural and migrant population. No significant differences were found in the prevalence of cognitive impairment between rural and migrant population. No case of dementia was reported from tribal population.
Lindesay, J., et al. (1997) (39)	297 aged ≥65 older adults living in UK (149 Gujarati and 148 native whites) Community setting	Cross-sectional study	Cognitive status: 1. CAMDEX 2. MMSE	Country of birth	Age, Education, Visual impairment, Ethnic group	Compared to native whites, Gujarati immigrants showed poorer orientation in time (day, year) and place (street), measured by MMSE. The proportion of cognitive impaired participants at all severities in the Gujarati immigrants were higher than in the native whites
Segers, K., et al. (2013) (40)	1058 new patients from the Memory Clinic in Brussels, Belgium (861 nonimmigrants, 106 European immigrants, and 91 non-European immigrants) Clinical setting	Cross-sectional study	Cognitive status: 1. MMSE Dementia: Medical record	Country of birth	N/A	Compared to nonimmigrants and non-European immigrants, European immigrants were more likely to have Parkinson related cognitive disorders. Compared to nonimmigrants and European immigrants, non-European immigrants showed poorer cognitive function measured by MMSE
Raina, S. K., et al. (2010) (41)	1856 aged ≥60 living in North India (Dogra population and migrant Kashmiri Pandit population) Community setting	Cross-sectional study	Cognitive status: 1. MMSE Dementia: Clinical evaluation	N/A	N/A	Compared to migrant Kashmiri Pandit population, the prevalence of dementia was significantly lower in the Dogra population
Kahana, E., et al. (2003) (42)	1720 aged ≥75 living in Ashkelon, Israel (659 Afro-Asian origin, 841 Europe-American origin) Community setting	Cross-sectional study	Dementia: DSM-III	Country of birth	Age, Education, Gender,	The prevalence of dementia was significantly higher in people of Afro-Asian origin than in people of Europe-American origin Ethnic origin was not a significant risk factor for dementia while controlling for all other factors
Livingston, G., et al. (2001) (43)	1085 aged ≥65 older adults living in UK (667 White British, 139 Irish, 71 Cypriot, 98 Black African/Caribbean, 60 immigrants from European countries, 50 immigrants from other countries) Community setting	Cross-sectional study	Dementia: The shortened version of the Comprehensive Assessment and Referral Evaluation (Short-CARE)	Country of birth Ethnicity	Age, Education, Gender, Income, Occupation, Drinking behavior, Chronic diseases (diabetes, stroke, CVD)	Compared to natives, the prevalence of dementia was higher in African-Caribbeans, after controlling for other factors Compared to natives, the prevalence of dementia was lower in individuals born in Ireland, after controlling for other factors
Touradj, P., et al. (2001) (45)	193 non-Hispanic Whites in the U.S. aged ≥65 (106 nonimmigrants and 87 immigrants) Community setting	Cross-sectional study	Cognitive status: 1. SRT 2. Benton Visual Retention Test (BVRT) 3. MMSE 4. WAIS-R 5. Identities and Oddities subtest of the Mattis Dementia Rating Scales (SRS Identities and Oddities subtest) 6. Boston Naming Test 7. Letter fluency: 8. Boston Diagnostic Aphasia Examination 9. Rosen Drawing Test 10. BVRT matching	U.S. born/foreign born Age at immigration	Age, Education, Gender	Compared to immigrants, nonimmigrants showed significantly higher scores on measure of abstract reasoning (WAIS-R), naming (Boston Naming Test), and fluency (letter and category fluency) Longer stay in the U.S. was associated with better score in WAIS-R.
Haan, M. N., et al. (2011) (46)	1789 aged ≥60 Mexican American in the U.S (827 first generation immigrants, 645 s generation, 67 third generation, 93 fourth generation) Community setting	Longitudinal study, follow-up period: 9 years	Cognitive status: 1. 3MSE 2. SEVLT	Country of birth Nativity of their parents and grandparents	Age, Education, Gender, Income, Occupation, Insurance, Childhood and adulthood SES, Chronic diseases (diabetes, stroke, hypertension)	Compared to first generation immigrants, second and third generation demonstrated better global cognitive function Compared to first generation immigrants, second generation demonstrated better short term memory Compared to first generation immigrants, the effect of life-time SES disadvantages on short term memory was greater in second generation immigrants.
Plitas, A., et al. (2009) (47)	142 aged ≥55 (76 Greek older adults, 66 Greek Australian) Clinical setting	Cross-sectional study	Cognitive status: 1. CAMDEX 2. MMSE	Country of recruitment	Age, Education, Gender	Greek Australians showed a significantly poorer performance on CAMDEX compared to Greek elderly Greek Australians showed a significantly poorer performance on MMSE compared to Greek elderly A significant Group × Gender interaction on both CAMCOG and MMSE were observed
Nielsen, T. R., et al. (2012) (48)	109 aged ≥50 (73 Turkish immigrants, 36 Danish) Community setting	Cross-sectional study	Cognitive status: 1. Recall of Pictures Test (RPT) 2. Clock Reading Test (CRT) 3. Supermarket Fluency (SF)	Country of birth	Age, Education, Acculturation	Turkish immigrants showed a significantly pooper performance on CRT and SF compared to Danish. No significant differences were found regarding RPT test Age, acculturation level were significantly association with test performances only among Turkish immigrants
Wilson, R. S., et al. (2005) (49)	6158 aged ≥65 living Chicago, United States (non-migrants and migrants) Community setting	Longitudinal study follow-up period: up to 6 years	Cognitive status: 1. MMSE 2. The East Boston Story: immediate and delayed recall 3. The Symbol Digit Modalities Test	County of birth	Age, Education, Gender, Childhood SES, Occupation, Birth county characteristics	Compared to residents born in Cook county, residents born elsewhere in the U.S showed lower global cognition score at baseline. Born in a higher socioeconomic level county was associated with a higher cognitive function. No significant associations were found regarding county of birth and rate of cognitive decline.
Graves, A. B., et al. (1999) (50)	1836 Japanese Americans: Issei (Japanese immigrants) Kibei (American-born but educated in Japan), and Nisei (American-born and educated) aged ≥65 in the U.S. Community setting	Longitudinal study, follow-up period: 2 years	Cognitive status: 1. CASI	Country of birth U.S./Japan educated Years living in Japan	Age, Education, Gender, Income, Acculturation, APOE ε 4,	Compared to Nisei, Issei and Kibei were less likely to experience cognitive decline during the 2-year follow-up period Longer years lived in Japan before age 18 was associated with less likelihood of cognitive decline
Yano, K., et al. (2000) (51)	3734 Japanese American men in the U.S. aged 71–93 (first and second generation) Community setting	Cross-sectional study	Cognitive status: 1. CASI	Country of birth Childhood years in Japan	Age, Education, Gender, Income, Occupation, Fluency in Japanese, Tofu and fish consumption, APOE ε 4	Longer childhood years in Japan was significantly associated with lower CASI score. Among participants who preferred to be testing in Japanese, longer childhood years in Japan was significantly associated with higher CASI score. Among participants who preferred to be tested in English, longer childhood years in Japan was significantly associated with lower CASI score
Stouten, L. H., et al. (2013) (52)	407 schizophrenia patients in the Netherland (157 Dutch, 138 1st generation immigrants, 112 2nd generation immigrants) Clinical setting	Cross-sectional study	Cognitive status: 1. Rey’s Auditory Verbal Learning task (RAVLT) immediate/delayed recall 2. Continuous Performance Task (CPT)	Country of birth	Age, Education, Gender, Physical functioning, Medication	Immigrants showed larger cognitive deficits in RAVLT (both immediate and delayed) and CPT comparing to the Dutch First generation Immigrants showed larger cognitive deficits in RAVLT (both immediate and delayed) and CPT comparing to the second generation Immigrants from Morocco, Turkey and other Non-Western countries demonstrated larger cognitive deficits in RAVLT immediate recall and CPT comparing to immigrants from other countries
Al Hazzouri, A. Z., et al. (2011) (53)	7042 aged ≥60 Mexicans and Mexican Americans (4687 Mexicans, 562 Mexican-return migrants, 908 Mexican-immigrants to the U.S., 871 Mexican-U.S. born) Community setting	Cross-sectional study using two large datasets	Cognitive status: 1. SEVLT 2. Three 8-word memory trails	Country of birth Current place of residency History of immigrating to the U.S.	Age, Education, Gender, Income, Insurance, Parents’ education, Chronic diseases (diabetes, stroke, hypertension, heart attack)	Compared to Mexicans, return migrants showed better cognitive function while both immigrants to the U.S. and U.S. born Mexicans showed poorer cognitive function Compared to Mexicans, the positive effect of parents’ education on cognitive function is greater in U.S born Mexicans

3MSE = Modified Mini-Mental State Examination

AD = Alzheimer’s disease

CAMDEX = the Cambridge Mental Disorders of the Elderly Examination

CASI = Cognitive Abilities Screening Instrument

DSM-III = Diagnostic and Statistical Manual of Mental Disorders, 3rd Edition

DSM-IV = Diagnostic and Statistical Manual of Mental Disorders, 4th Edition

ICD-10 = the 10th revision of the International Statistical Classification of Diseases and Related Health Problems

MCI = Mild Cognitive Impairment

MMSE = Mini-Mental State Examination

NIDR = National Institute of Dental Research

NINCDS-ADRDA = National Institute of Neurological, Communicative Disorders, and Stroke-Alzheimer’s Disease and Related Disorders Association

SES = Socioeconomic status

Short-CARE = shortened version of the Comprehensive Assessment and Referral Evaluation

SEVLT = Spanish English Verbal Learning Test

SRT = Simple Reaction Time

WAIS-R = Wechsler Adult Intelligence Scale-Revised

Only 9 of the 25 articles applied longitudinal design. The follow-up period of the longitudinal studies varied from 1 year [30] up to 23 years [31]. Nine studies used a secondary data analysis approach, and 4 of these analyzed the data from the Hispanic Established Populations for the Epidemiologic Study of the Elderly [32–35]. Sample size also ranged from small (*N* = 81) [36] to large (*N* = 12,008), with the latter including samples from several large datasets, such as the Sacramento Area Latino Study on Aging and the Mexican Health and Aging Study [37].

### Measures of cognitive function

Assessment of cognitive function included use of standard clinical diagnostic criteria of dementia, information from medical records, epidemiological screening measures, and a variety of neuropsychology measures. Four studies applied a two-stage cognitive function assessment in which participants were initially screened and then received clinical evaluation of dementia [30, 38–41].

Two studies applied standard diagnostic criteria for dementia DSM-III [31, 42] and one used DSM-IV and ICD-10 [28]. Other criteria included NINCDS-ADRDA for Alzheimer’s disease [28, 30, 36] and NINDS-AIREN for vascular dementia [28, 36]. Only one study used the shortened version of the Comprehensive Assessment and Referral Evaluation (Short-CARE) to identify persons with dementia [43]. Three studies did not specify the criteria that was used for dementia diagnosis [38, 40, 41].

The most widely used single measure of cognitive status is the MMSE, and this was used in 14 studies [44], one of which adapted the orientation questions from the MMSE as part of the neuropsychology measures [45]. One study from India used the Hindi Mental State Examination, a modified version of the MMSE to make the instrument culturally appropriate [38]. At times other instruments including the Modified Mini Mental Status Exam (3MSE) [36, 46] and the Cambridge Mental Disorders of the Elderly Examination (CAMDEX) [28, 39, 47] were used to measure individuals’ global cognitive function. Six studies applied a substantial cognitive battery to assess different aspects of cognitive function [31, 45, 48, 49]. All the measures used in the 25 studies, including both clinical diagnostic tools and epidemiological screening measures, were shown to be valid and reliable.

### Measures of migration

Among the 22 studies that focused on immigration, 21 identified immigrants based on whether they were born in the receiving country. Only one study conducted in Canada defined immigrants as those whose first language was neither English nor French [30]. Nineteen studies included information on country of birth, 6 of which asked participants their age at immigration or length of stay in the receiving country [29, 31, 32, 45, 50, 51].

Three studies focused on internal migrants. Two studies conducted in India did not specify the definition of migrants [38, 41]. The third study defined migrants to be individuals who lived in Cook County, Illinois, but were born elsewhere [49]. None of the 25 articles assessed the reason for migration.

### Covariates included in the analyses

Twenty-one articles included some of the sociodemographic characteristics (e.g. age, gender, and education) as covariates in the analyses. Other common covariates included functional disability [32, 34, 52], chronic disease [32–35, 43, 46, 52, 53], health behaviors [32, 43, 51], depressive symptoms [32–34], and early- and middle-life socioeconomic conditions [35, 46, 49]. Two studies included environmental factors at neighborhood [35] or county level [49]. The genetic biomarker APOE ε 4 was also considered in two studies [50, 51].

### Findings

#### Comparisons between immigrants and residents in hosting countries

Nine studies compared first-generation with second-generation immigrants regarding their cognitive function, and one of those studies also included third- and fourth-generation immigrants [46]. Four of them indicated no significant differences between first-generation and second-generation immigrants in cognitive function at baseline or the rate of cognitive decline [34, 35, 51, 53]. This result was contradicted by other studies that showed differences in cognitive function between first- and future-generation immigrants [32, 33, 46, 50, 52]. Further, findings were inconsistent among these five studies. Four studies showed that first-generation immigrants demonstrated poorer cognitive function [33, 46, 52] than second- and even the third-generation immigrants. However, one study indicated that first-generation Japanese immigrants were less likely to experience cognitive decline than the second-generation immigrants [50]. Another study found the association between immigration and cognitive function varied by gender and age at immigration [32]. Hill et al. [32] showed that male Mexican American immigrants who migrated to the United States in their middle age showed both better cognitive function at baseline as well as a slower decline rate compared to U.S-born Mexican American men. Immigration status and age at immigration were not significantly associated with both baseline cognitive functioning and the cognition trajectories among the female population [32].

Four studies found that immigrants had poorer cognitive function compared to individuals in hosting countries that are native-born residents [39, 40, 45, 48, 52]. One study showed that a higher proportion of immigrants scored as “cognitively impaired” measured by the CAMDEX [39]. Still, two studies showed no difference in cognitive function between immigrants and native-born residents [29, 30].

Four studies examined the association between immigration and incidence of dementia [28, 30, 36, 43]. Two studies conducted in the UK reported a higher prevalence of dementia among African-Caribbean immigrants than those born in the UK [28, 43]. The other two studies that focused on persons with dementia showed no significant association between immigrant status and age of dementia diagnosis [30, 36]. In addition, these two studies explored the interaction between bilingualism and immigration and their possible effects on age of dementia diagnosis. Lawton et al. [36] found no significant interaction effect while the other study showed that native English monolinguals were diagnosed with dementia at a later age than immigrant monolinguals [30].

#### Comparisons between immigrants and residents in sending countries

Three studies compared immigrants versus residents in their sending countries [37, 47, 53]. Two cross sectional studies compared local Mexican residents to Mexicans who immigrated to the United States; the results of these two studies contradicted each other. One cross-sectional study that used several different data sources found that the prevalence of cognitive impairment was similar among Mexican immigrants and Mexicans [37]. However, the other study demonstrated that compared to Mexicans, both immigrants to the United States and U.S-born Mexicans had poorer cognitive function [53]. This study also found that return migrants from the United States showed better cognitive function than local residents in Mexico [53]. The third study conducted in Australia also reported poorer cognitive function among Greek Australians compared to their counterparts living in Greece [47].

#### Comparisons across immigrant groups

Four studies made comparisons across different immigrant populations in the same hosting country [29, 31, 40, 42]. Two of them found no significant difference in cognitive function among immigrants from various countries [29, 42]. But these results were contradicted by two other studies. One study conducted in Belgium showed that compared to European immigrants, non-European immigrants had poorer cognitive function measured by the MMSE and were more likely to have Parkinson’s-related cognitive disorders [40]. The other study found immigrants in the U.S. from the Dominican Republic/Puerto Rico showed poorer initial performance on memory and language tests, but not the rate of change in any cognitive domains between Dominican Republic/Puerto Rican immigrants and immigrants from other countries [31].

#### Comparisons among internal migrants and non-migrants

Three studies examined the association between internal migration and cognitive function, two of which indicated migrants had poorer cognitive function than non-migrants [41, 49]. Another study from India showed that urban residents had a higher prevalence of cognitive impairment than rural and migrant population in India [38]. However, no statistically significant differences were found in the prevalence of cognitive impairment between rural and migrant population [38].

#### Findings from longitudinal studies

Using longitudinal data, several studies found no significant differences in the rate of cognitive decline between migrants and non-immigrants [29, 30, 34, 35, 49], or between immigrants from different countries of origin [29, 31]. However, two studies demonstrated a protective effect of migration on cognitive decline [32, 50].

#### Assessment of the quality of the eligible studies

Each of the studies was assessed using a set of criteria developed earlier, including sample selection, sample size, validated assessment of outcome, attrition, conflict of interests report, and analytic approach [54, 55]. The evaluation of each article suggested methodological deficiencies in this area of research. The most problematic aspects included baseline incomparability (e.g. significant differences in sample characteristics between migrants and non-migrants, *n* = 25), inadequate assessment of cognitive function (e.g. only used a screening tool such as MMSE to measure cognitive function, *n* = 17), lack of longitudinal studies (*n* = 16), inadequacy in addressing incomplete data (e.g. no information on missing data management, *n* = 16), and high dropout rate (e.g. dropout rate = 35%, *n* = 19). Therefore, the current evidence was low quality and therefore systematic conclusions cannot be made. Detailed results are available from the authors upon request.

## Discussion

This is the first systematic review that examined the association between migration and cognitive function. A total of 25 articles were identified with 22 immigration studies and another 3 studies focused on internal migration. The results from the review showed inconsistent associations between migration and cognitive function. For example, some studies found a significant negative association, albeit relatively weak, between migration and cognitive function; but other studies found no association. This may largely due to the wide variation in the study design, sample size, sample characteristics, and measures across studies. More importantly, it would be essential to include the reasons for migration because this may have implications for individual’s financial status, social support, sense of well-being, and health status including cognitive function. Because of the inconclusive finding, we didn’t discuss the potential implications for clinical practices and health policies.

Most studies compared migrants with people in hosting countries and some demonstrated that immigrants had poorer cognitive function. It is possible that the disparities in cognitive function between migrants and non-migrants could be partially explained by low level of acculturation, such as poor language skills. Several studies found that immigrants who had better acquisition of the language that the cognition test was conducted in (i.e. the language used in the hosting countries) showed better cognitive function [31, 33, 47, 48, 51]. Language is associated with some domains of cognitive function. When studying cognitive function among immigrant population, we need to be aware whether the proficiency of certain language may bias the test results. Thus, it would even be more important to use longitudinal studies to compare the changes in cognitive function among immigrant population, in comparison with the native-born population in hosting countries.

Another possible explanation is SES pathway as we described in the introduction. Immigrants are likely to have the disadvantages in SES, especially Hispanic immigrants, compared with the native-born population. Several studies reported that immigrants tended to have a lower level of education and more financial strains than residents in hosting countries [32, 33, 46, 48, 53]. In addition, migrants didn’t necessarily have better SES compared to non-migrants in the sending countries [47, 53]. It might due the incomparability in SES measures such as the quality of education and wages standards between hosting and sending countries [47, 53]. Previous research showed strong association between SES and cognitive function [56]. Education has been shown to positively influence an individual’s cognitive function in late life [11]. Moreover, SES benefits may lead to improvement in health service utilization and better care of chronic conditions, which are associated with an individual’s cognitive function [12].Therefore, the SES disadvantage may partially contribute to poorer cognitive function among immigrant populations. However, the SES disadvantage pathway may only apply for the Hispanic immigrant populations. Studies that focused on immigrants from Japan showed no significant difference in SES between Japanese immigrants and Japanese Americans [50, 51]. One study that compared Mexican-return migrants with Mexicans found better SES among Mexican-return migrants, which might be explained by the observed SES advantages among Mexican-return migrants [53].

In addition, as we expected, some studies suggested that migration-related life style changes may serve as mediators that link migration and cognitive function. For example, studies showed Japanese immigrants who preferred traditional Japanese diet were more likely to have better cognitive function [50, 51]. This may because traditional Japanese diet was associated with fewer chronic diseases that were risk factors of cognitive decline [51]. Similarly, one study that compared Mexicans versus Mexican Americans found better cognitive function among Mexicans [53]. One explanation is that Mexicans showed healthier life styles and better chronic disease profiles, which are protective against cognitive decline [53].

Some other studies argue that migrants who come from unfavorable early-life socioeconomic positions may have residual negative effects on later-life cognitive function [32, 35, 42, 46, 49, 52]. However, results from limited studies controlled for early-life socioeconomic status (three out of 25), and studies that compared immigrants and non-migrants from sending countries are inconsistent. Therefore, whether migration could buffer the negative effects of early-life disadvantage on cognitive function is inconclusive.

With the aging population increasing, the burden of cognitive impairment and dementia is expected to increase. In the meantime, the migrant population, especially internal migrants in developing countries, is increasing dramatically. Research on the association between migration and cognitive function is still in the early stages, which results in many potential implications for future research.

To better examine the association between migration and cognitive function, more comprehensive measures of the migration process are needed. Most of previous studies defined migration status based on birth place and current living place, which is a rather crude method. Only 6 studies measured age at immigration or length of stay in the receiving country [29, 31, 32, 45, 50, 51]. However, no studies included information on participants’ reasons for migration (e.g., education, family reunion, economic condition, and political asylum). Refugees and people who migrated for the purpose of better education are likely to have different SES, psychosocial distress, and/or health status which may affect the level of cognitive function. Therefore, without knowing the whole picture of the migration process, it would be difficult to identify the specific aspects of migration that might influence an individual’s cognitive function. Therefore, including more detailed information on the migration history is necessary for future research. In addition, qualitative studies would be useful because they allow researchers to use in-depth interviews to capture the migration process of each individual, and the resulting data could supplement quantitative studies.

In addition, future research should include more sensitive, valid and culturally appropriate clinical measures of cognitive impairment and dementia. Only 8 articles used validated clinical instrument to measure cognitive function and the MMSE [44] is the most frequently used measure in literature especially among population-based studies. However, research has shown the ceiling and floor effects of this instrument in detecting cognitive decline [57, 58]. Additional tests are recommended to detect possibility of mild cognitive impairment (MCI) or dementia. In addition, future studies should apply culturally specific versions of cognitive function measures to ensure their reliability and validity. Participants who are immigrants should be tested in their preferred language. Although the MMSE has multiple language versions, the comparability among them remains unknown. Therefore, future research should consider using measures such as the Montreal Cognitive Assessment that are sensitive to changes in cognitive function and could accommodate certain linguistic and cultural differences [59].

Evaluation of the study quality suggested that there were methodological deficiencies in the current literature about migration and cognitive function. Only 9 studies applied longitudinal design [29–32, 34, 35, 46, 49, 50], 9 adequately addressed incomplete data [30–32, 45, 46, 48–50, 53], and 6 had a dropout rate < 30% [28, 31, 32, 46, 49, 50]. These deficiencies are likely to produce biased results and limit the generalizability of the study findings. From a life-course perspective, the impact of migration or migration related changes can accumulate throughout the life-course. We also noticed that studies with higher quality tend to find differences in cognitive function (either baseline or slope differences) between migrants and non-migrants. Therefore, more longitudinal studies using validated measures and appropriate control are essential to establish a strong association between migration and cognitive function.

A large variety of covariates were reported in the previous studies, including demographic characteristics, health behaviors and health status. Still, only adjusting for these covariates may not be sufficient. Results from our quality assessment indicated that migrants and non-migrants had relatively different characteristics. It is possible that some differences in level of cognition between migrants and non-migrants are likely to be explained by confounding variables other than migration status. Thus, using more sophisticated statistical methods should be encouraged to adjust for confounding factors. For example, using propensity scores, researchers are able to balance the distributions of observed covariates between treatment conditions (e.g. rural residents and rural-to-urban migrants) so that a direct comparison between matched treatment conditions becomes valid [60]. Another solution is to use instrumental variable analysis to minimize the unmeasured cofounding factors [61]. Additionally, several other factors that are associated with cognitive function and migration were rarely included in the literature. For example, migrants are likely to experience changes in psychosocial factors such as social support and social network during and after the migration process [23]. Some studies found social isolation to be a risk factor for cognitive impairment and cognitive decline [26, 62, 63], and many migrants face the challenge of decreasing social network. In addition, studies have shown that stressful life events are associated with increased dementia risk [64, 65]. However, none of the included studies controlled for these psychosocial factors, which should be considered in future research.

Only three studies have been conducted to assess the cognitive function among internal migrants. Given the tremendous increase in rural-to-urban migrant population globally, it is important to examine the association between internal migration and cognition using longitudinal data. Research in this area will provide new knowledge on health disparities particularly among rural residents and rural-to-urban migrants. Comparisons across three groups (i.e., rural residents, urban residents, and rural-to-urban migrants) will allow researchers to study the environmental differences and health disparities between rural and urban areas and understand the impact of rural-to-urban migration on health status in later life. If such disparities exist, research that assesses health disparities should not only compare health status differences between rural and urban residents, but also consider the migrant group.

## Conclusion

Overall, the evidence from current studies regarding the association between migration and cognitive function is weak and inconclusive. Findings were inconsistent across studies, and the association ranged from a negative association, to no association, to a positive association (albeit, in only one study). The quality of current literature suffered from methodological deficiencies, with limited studies applying longitudinal design, using validated outcome measures, addressing potential selection bias adequately. Additional research is needed to examine the linkages using more rigorous study design and validated instruments.

## Abbreviations

3MSE: Modified Mini-Mental State Examination
AD: Alzheimer’s disease
CAMDEX: the Cambridge Mental Disorders of the Elderly Examination
CASI: Cognitive Abilities Screening Instrument
DSM-III: Diagnostic and Statistical Manual of Mental Disorders, 3rd Edition
DSM-IV: Diagnostic and Statistical Manual of Mental Disorders, 4th Edition
ICD-10: the 10th revision of the International Statistical Classification of Diseases and Related Health Problems
MCI: Mild Cognitive Impairment
MMSE: Mini-Mental State Examination
NIDR: National Institute of Dental Research
NINCDS-ADRDA: National Institute of Neurological, Communicative Disorders, and Stroke-Alzheimer’s Disease and Related Disorders Association
SES: Socioeconomic status
SEVLT: Spanish English Verbal Learning Test
Short-CARE: shortened version of the Comprehensive Assessment and Referral Evaluation
SRT: Simple Reaction Time
WAIS-R: Wechsler Adult Intelligence Scale-Revised
